# Exploring the Feasibility of Pipeline Embolization Device Compared With Stent-Assisted Coiling to Treat Non-saccular, Unruptured, Intradural Vertebral Artery Aneurysms

**DOI:** 10.3389/fneur.2019.00275

**Published:** 2019-03-26

**Authors:** Yupeng Zhang, Fei Liang, Yuxiang Zhang, Peng Yan, Shikai Liang, Chao Ma, Chuhan Jiang

**Affiliations:** ^1^Department of Interventional Neuroradiology, Beijing Neurosurgical Institute and Beijing Tiantan Hospital, Capital Medical University, Beijing, China; ^2^Department of Neurosurgery, School of Clinical Medicine, Beijing Tsinghua Changgung Hospital, Tsinghua University, Beijing, China

**Keywords:** pipeline embolization device, non-saccular aneurysm, vertebral artery aneurysm, stent-assisted coiling, posterior circulation

## Abstract

**Object:** The pipeline embolization device (PED) has been used to treat non-saccular, unruptured, intradural vertebral artery aneurysms at some institutions. However, there is an absence of large controlled studies validating the feasibility of this treatment. This study aimed to explore the therapeutic feasibility of PED by comparing PED treatment with controlled stent-assisted coiling (SAC).

**Materials and Methods:** Thirty-two PED procedures were matched in a 1:2 manner with 64 SAC procedures based on patient age, sex, aneurysm size, and aneurysm location. Technical factors, procedural complications, angiographic results, and clinical outcomes were analyzed and compared.

**Results:** There was no statistically significant difference in technical factors and procedural complications between the two groups (PED vs. SAC, 9.4 vs. 4.7%, *P* = 0.397). In multivariate analysis, smoking and therapeutic modality were identified as independent predictors of occlusion. Smoking was a risk factor for aneurysm obliteration [hazard ratio (HR) 0.53; 95% confidence interval (CI), 0.31–0.89; *P* = 0.018]. Aneurysms treated with PED were more likely to achieve obliteration over time compared with aneurysms treated with SAC (HR 2.97; 95% CI, 1.79–4.93; *P* < 0.001). The rate of favorable clinical outcomes (modified Rankin Scale (mRS), 0–2) was similar between the two groups (PED vs. SAC, 100 vs. 96.9%, *P* = 0.551). In the SAC group, one patient had neurological deficit with an mRS of four at the latest follow-up. There was no mortality in either group.

**Conclusions:** The PED and SAC groups showed similar technical factors, procedural complications, angiographic results, and favorable clinical outcomes. Aneurysms treated with PED were more prone to obliteration over time than aneurysms treated with SAC. These outcomes suggest, based on short-term follow-up, PED is a safe and feasible strategy for the treatment of non-saccular, unruptured, intradural vertebral artery aneurysms.

## Introduction

The pipeline embolization device (PED; ev3/Covidien Neurovascular, Irvine, CA, United States) is one of the most commonly used flow diverters, initially approved by the Food and Drug Administration (FDA) in 2011. Since the introduction of the PED, it has gained popularity in the treatment of anterior circulation intracranial aneurysms, with high rates of obliteration, and low complication rates (1–4). Previous studies have also reported that the PED can be used safely for posterior circulation aneurysms and can obtain favorable therapeutic outcomes with complete occlusion rates of 78.6–96% (5–7). However, the feasibility of PED for previously untreated, unruptured, non-saccular, intradural vertebral artery aneurysms has not been clearly demonstrated. Although using PED to treat these aneurysms has yielded a complete obliteration rate of 66.7–100% in some small previous studies (8–10), comparison of PED with the commonly used stent-assisted coiling (SAC), trapping, or coiling has not been performed in previous studies. In this study, we compared PED with SAC to explore whether the indications for PED can be extended to include previously unruptured, non-saccular, intradural vertebral artery aneurysms.

## Materials and Methods

### Population

Thirty-two consecutive unruptured, non-saccular, intradural vertebral artery aneurysms treated with PED (11/2015-11/2016) at our institution were identified from our clinical database. To achieve more accurate outcomes, every PED procedure was matched to two control SAC procedures (3/2015-7/2017) on the basis of patient age, sex, aneurysm size, and aneurysm location. Procedures were excluded from this cohort study if the patients' aneurysm(s) had previously ruptured, had been treated, or were saccular in morphology. All of the included non-saccular aneurysms were fusiform aneurysms. The indications for treatment were aneurysms with high risk of rupture and patients with clinical symptoms (headache, dizziness, numbness of limb, vomiting, dysphagia; Table 1). And all patients included in this retrospective study signed informed consent forms before treatment and the study was approved by our ethics committee.

**Table 1:** Patient and aneurysm characteristics.

**Characteristics**	**PED group**	**SAC group**	***P*-value**
Aneurysms' number	33	65	—
Patients' number	30	64	—
Procedures' number	32	64	—
Females, number (%)	6 (18.8)	7 (10.9)	0.348
Smoking, number (%)	15 (46.9)	27 (42.2)	0.670
Age (years), median (IQR)	51 (47–58)	53 (46.8–59)	0.367
Indications for treatment, number (%)			—
Aneurysms with high	7 (21.9)	24 (37.5)	
risk of rupture			
Headache	18 (56.3)	18 (28.1)	
Dizziness	13 (40.6)	21 (32.8)	
Numbness of limb	2 (6.3)	3 (4.7)	
Vomiting	5 (15.6)	1 (1.6)	
Dysphagia	0 (0)	1 (1.6)	
Aneurysms location, number (%)			0.567
PICA involved	4 (12.5)	12 (18.8)	
PICA not involved	28 (87.5)	52 (81.2)	
Aneurysms' size (mm), median (IQR)	11 (9.6–13.9)	11.6 (8.4–15.0)	0.635
Parent artery stenosis (>50%), number (%)	3 (9.4)	9 (14.1)	0.745
Baseline mRS, number (%)			1
mRS 0-2	31 (96.9)	62 (96.9)	
mRS 3-5	1 (3.1)	2 (3.1)	

### Endovascular Treatment

Patients were premedicated with clopidogrel (75 mg/day) and aspirin (100 mg/day) orally 5 days before endovascular treatment. Thromboelastograph testing (TEG) was used to test the inhibition rate of platelet activity. The required inhibition rate was 30–80% for adenosine diphosphate (ADP) and >70% for arachidonic acid (AA). The treatment modalities (PED or SAC) were decided by more than two interventional neurologists based on the patient demographic, patient symptom, and aneurysm characteristic. All endovascular procedures were performed under general anesthesia with systemic heparinization and mainly performed by tree experienced and skillful interventionalists. A biaxial system with the combination of a 6-F guiding catheter (Codman, Raynham, MA, United States) and Marksman (EV3, Irvine, CA, United States) was adopted to deploy the PED. Once the PED reached the scheduled position, it was released carefully by a combination of withdrawing the Marksman catheter and advancing the delivery wire. Three types of stents [Low-prole Visualized Intraluminal Support (LVIS) (MicroVention, Tustin, CA, United States), Enterprise (Cordis Neurovascular, Miami Lakes, FL, United States), and Neuroform EZ (Boston Scientific, Fremont, CA, United States)] were used in the stent-assisted coiling group. The coils were packed in the aneurysm by microcatheter jailing. When the stent did not fully appose to the parent vessel wall, balloon angioplasty would be performed. Clopidogrel (75 mg/day) was discontinued 3 months after the procedure and aspirin (100 mg/day) was continued for 6 months after the procedure, if the aneurysms were complete occlusion and the patients had not any complications.

### Outcome Management

The angiographic and clinical outcomes of the 32 PED procedures and 64 SAC procedures matched for patient age, sex, aneurysm size, and aneurysm location were compared. Immediate aneurysm occlusion rates were denoted as percentages after the procedures and transformed into a binary variable: complete occlusion (100%) and incomplete occlusion (<100%). Stent migration, insufficient opening (>50%), foreshortening of the stent, and rupture of parent artery were defined as technical events. Angiographic follow-up [digital subtraction angiography (DSA) and computed tomographic angiography (CTA)] was scheduled at 3 to 6 months and 1–2 years postoperatively. Angiographic outcomes were evaluated by the Raymond-Roy occlusion classification as follows: Raymond Class I (complete obliteration), Raymond Class II (residual neck), and Raymond Class III (residual aneurysm) (11). Clinical outcomes at the latest available follow-up were classified by the modified Rankin Scale (mRS), and mRS under three was defined as a favorable outcome.

### Statistical Analysis

Statistical analysis was performed with R 3.4.3 (R Foundation for Statistical Computing, Vienna, Austria). Data were analyzed on a per-procedure basis. For continuous variables, data that obeyed normal distribution are presented as mean and standard deviation. Data that did not obey normal distribution are presented as median and inter-quartile range. For categorical variables, data are presented as the absolute value followed by percentage. Univariate Cox regression analysis was performed to test covariates predictive of the time-dependent outcomes, namely, the complete occlusion of aneurysms with parent artery patency. Variables entered in the analysis included age, sex, smoking, aneurysm size, PICA arising from the aneurysms, parent artery stenosis, immediate occlusion, and treatment modality. Factors predictive in univariate analysis (*P* < 0.10) were further selected for multivariate Cox regression analysis. Difference in time-to-endpoint between the two groups was illustrated by the Kaplan-Meier curve and the log-rank test was applied to calculate the statistical significance. Variables were compared between groups using the Mann-Whitney *U*-test for numerical variables and the Chi-squared test or Fisher exact test for categorical variables. Statistical significance was defined as *P* < 0.05.

## Results

### Patient Characteristics

The percentage proportion of female patients was 18.8 in the PED group vs. 10.9 in the SAC group (*P* = 0.348). Patients with a history of smoking accounted for 46.9% in the PED group vs. 42.2% in the SAC group (*P* = 0.670). Median patient age was similar in the PED group [51 years (47–58)] and the SAC group [53 years (46.8–59)] (*P* = 0.367). Median aneurysm size was 11 mm (9.6–13.9) in the PED group vs. 11.6 mm (8.4–15.0) in the SAC group (*P* = 0.635). The posterior inferior cerebellar artery (PICA) was involved in 4 (12.5%) procedures in the PED group and 12 (18.8%) procedures in the SAC group (*P* = 0.567). There was no obvious difference between the PED group and SAC group in terms of parent artery stenosis (>50%), with 3 (9.4%) in the PED group vs. 9 (14.1%) in the SAC group (*P* = 0.745). The rate of baseline mRS of 0–2 was similar between groups, with 31 (96.9%) vs. 62 (96.9%) in the PED and SAC groups, respectively. All patient characteristics were matched between the two groups and are summarized in Table 1.

### Aneurysm Treatment

Patients with aneurysms with an irregular morphology and clinical symptoms (headache, dizziness, numbness of limb, vomiting, and dysphagia) were defined as having indications for treatment. Thirty-six PEDs were used to perform the 32 operations, and in 28 (87.5%) cases one PED was implanted. Balloon angioplasty was performed in one procedure in the SAC group. The immediate complete occlusion rate of the SAC group was 64.1%. There was no difference in procedure length between the two groups (*P* = 0.386).

### Complications

**Figure 1:**
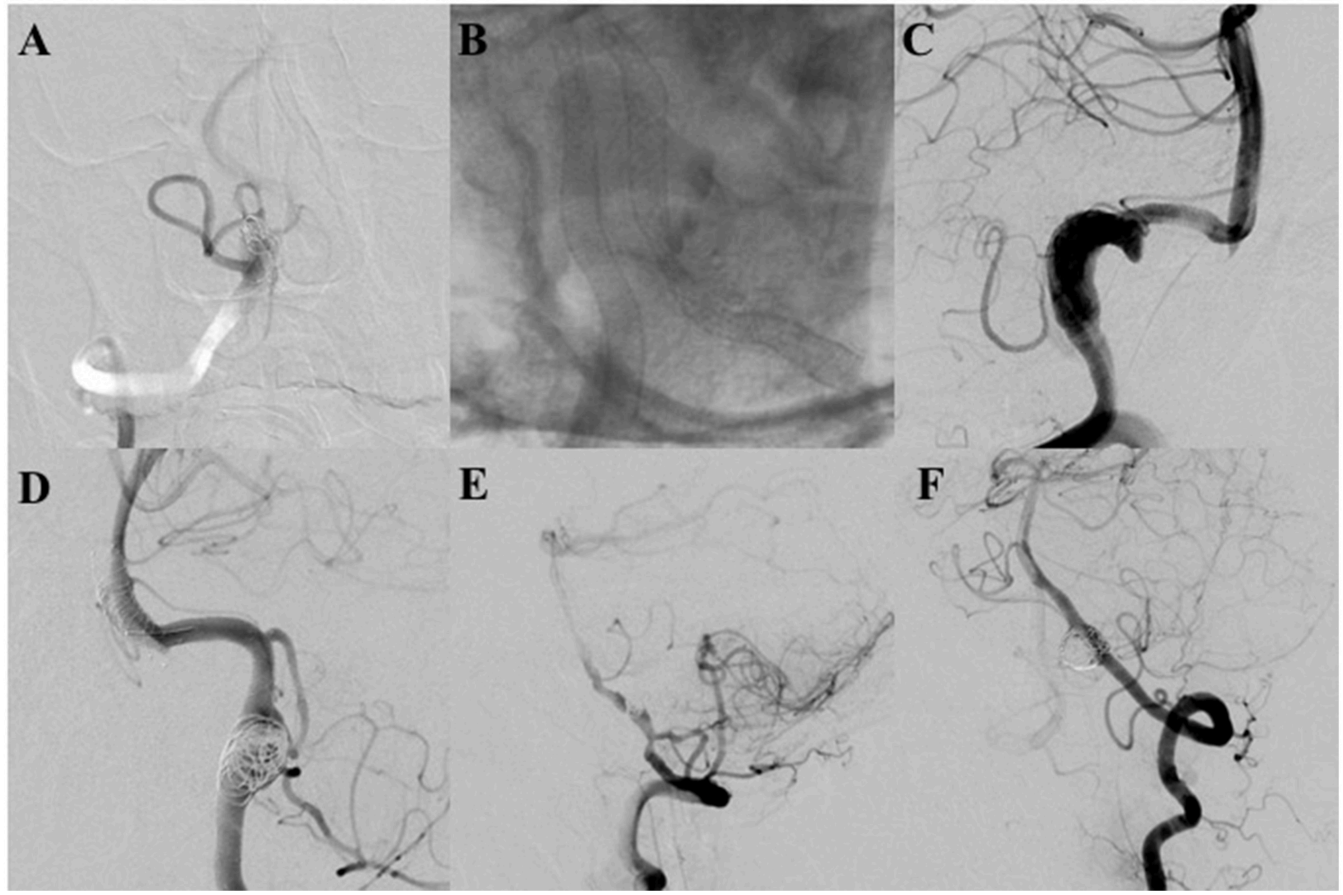
**(A,B)** The pipeline embolization device (PED) was not fully deployed in two patients because of stenosis of the parent artery, which caused parent artery occlusion. Fortunately, both patients were asymptomatic with patency of the covered posterior inferior cerebellar arteries (PICAs) at final angiographic follow-up. **(C)** Controlled contrast shows foreshortening of the PED that occurred in the procedure; the PED did not cover the target aneurysms. An additional PED was deployed. The aneurysm is completely occluded with parent artery patency at final angiography. **(D)** An LVIS stent shows migration to the basilar trunk artery during the procedure. Fortunately, the patient had no neurologic deficient. Angiography demonstrates that the aneurysm was completely occluded with parent artery patency. **(E)** The LVIS is not fully deployed in the patient due to stenosis of the parent artery. Final follow-up angiography demonstrates that the aneurysm was completely occluded with parent artery occlusion. The patient did not have any neurological deficit with the covered PICA patency. **(F)** The V3 segment artery was ruptured spontaneously. Final angiography demonstrates parent artery occlusion at the rupture site.

Three patients had peri-procedural complications (subarachnoid hemorrhage, minor ischemic stroke, and embolus formation) in the PED group compared to three patients (two minor ischemic stroke and one major ischemic stroke) in the SAC group (*P* = 0.397). Technical events occurred in 3 (9.4%) procedures (two insufficient opening of stent (>50%), one foreshortening of stent) in the PED group vs. 3 (4.7%) procedures (one migration of stent, one insufficient opening of stent (>50%), one rupture of parent artery) in the SAC group (*P* = 0.397; Figure 1). As described in Table 2, delayed complication (parent artery occlusion, PAO) occurred in four patients including 2 (6.7%) patients in the PED group vs. 2 (3.9%) patients in the SAC group (*P* = 0.625).

**Table 2:** Outcomes.

**Parameter**	**PED group**	**SAC group**	***P*-value**
Immediate occlusion rate, number (%)			< 0.001
Complete occlusion (100%)	0 (0)	41 (64.1)	
Incomplete occlusion (< 100%)	32 (100)	23 (35.9)	
Periprocedural complications, number (%)	3 (9.4)	3 (4.7)	0.397
Subarachnoid hemorrhage	1 (3.1)	0 (0)	
Minor ischemic stroke	1 (3.1)	2 (3.1)	
Major ischemic stroke	0 (0)	1 (1.6)	
Embolus formation	1 (3.1)	0 (0)	
Technical event, number (%)	3 (9.4)	3 (4.7)	0.397
Migration of stent	0 (0)	1 (1.6)	
Insufficient opening of stent	2 (6.3)	1 (1.6)	
(>50%)			
Foreshortening of stent	1 (3.1)	0 (0)	
Rupture of parent artery	0 (0)	1 (1.6)	
Procedure length (min), median (IQR)	95 (79.5–120)	90 (69–120)	0.386
Post-treatment mRS score, number (%)			1
0–2	31 (96.9)	62 (96.9)	
3–5	1 (3.1)	2 (3.1)	
Latest imaging follow-up available, number (%)	30 (93.8)	51 (79.7)	0.083
Follow-up occlusion rate, number (%)			0.118
Raymond class I	27 (90.0)	37 (72.5)	
Raymond class II	1 (3.3)	10 (19.6)	
Raymond class III	0 (0)	2 (3.9)	
Parent artery obliteration	2 (6.7)	2 (3.9)	
Patency of covered PICA, number (%)	10 (100)	27 (100)	—
Delayed complications, number (%)			0.625
Parent artery occlusion	2 (6.7)	2 (3.9)	
Kaplan-Meier Occlusion time (Median days)	165	248	< 0.001
Follow up mRS, number (%)			0.551
0–2	32 (100)	62 (96.9)	
3–5	0 (0)	2 (3.1)	
Morbidity	0 (0)	1 (1.6)	1
Mortality	0 (0)	0 (0)	—

### Angiographic Outcomes

Angiographic outcome data were available for 30 (93.8%) procedures in the PED group vs. 51 (79.7%) procedures in the SAC group (*P* = 0.083). The median follow-up time was 4.9 (3.3–6.7) months in the PED group vs. 6.8 (6.1–8.4) months in the SAC group (*P* < 0.001). All the covered PICAs were patent at the latest angiographic follow-up.

In univariate analysis, smoking (hazard ratio (HR) 0.60; 95% confidence interval (CI), 0.36–1.00; *P* = 0.050) and modality (PED vs. SAC) (HR 2.74; 95% CI, 1.66–4.53; *P* < 0.001) were identified as predictive factors for aneurysm obliteration. Multivariate analysis revealed that the aneurysms of the PED group were more likely to occlude than the aneurysms of the SAC group over time (HR 2.97; 95% CI, 1.79–4.93; *P* < 0.001); smoking was the risk factor associated with aneurysm obliteration (HR 0.53; 95% CI, 0.31–0.89; *P* = 0.018; Table 3).

**Table 3:** Cox analysis outcomes.

**Parameter**	**Hazard ratio**	**95%CI**	***P*-value**
**Univariate analysis**			
Age (>60 years)	1.09	0.54–2.21	0.814
Male gender	0.95	0.47–1.93	0.885
Smoking	0.60	0.36–1.00	0.050
Aneurysms' size	0.97	0.92–1.03	0.342
PICA arising from aneurysms	0.67	0.32–1.41	0.291
Parent artery stenosis (>50%)	0.60	0.24–1.50	0.278
Immediate occlusion	0.78	0.47–1.29	0.331
Modality (PED vs. SAC)	2.74	1.66–4.53	< 0.001
**Multivariate analysis**			
Smoking	0.53	0.31–0.89	0.018
Modality (PED vs. SAC)	2.97	1.79–4.93	< 0.001

**Figure 2:**
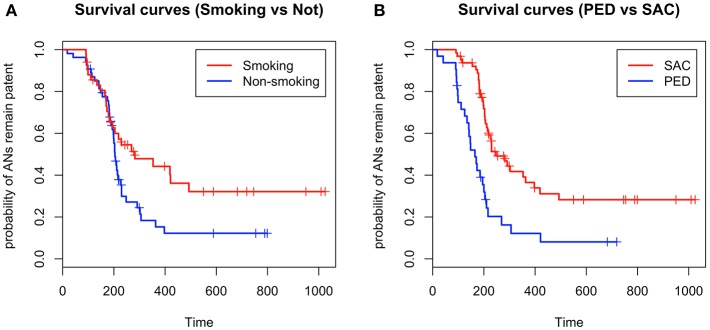
**(A)** Kaplan-Meier survival curves indicate that smoking might prolong the aneurysm obliteration time, **(B)** and that patients treated with the pipeline embolization device have a shorter interval to obliteration than patients treated with stent-assisted coiling (+ in survival curves refers to censored values).

The median probability of aneurysm obliteration (i.e., time to obtain obliteration in 50% of aneurysms) was evaluated by Kaplan-Meier analysis. The median probability of aneurysm obliteration was 204 days in the non-smoking group vs. 282 days in the smoking group (*P* = 0.048). The median probability of aneurysm obliteration was 165 days in the PED group vs. 248 days in the SAC group (*P* < 0.001). The outcomes are described by Kaplan-Meier survival curves in Figure 2.

### Clinical Outcomes

All final clinical outcomes were available for analysis. The median follow-up time was 7.9 (6.6–23.0) months in the PED group vs. 6.2 (4.7–7.6) months in the SAC group (*P* < 0.001). Favorable clinical outcomes occurred in 32 (100%) procedures in the PED group vs. 62 (96.9%) procedures in the SAC group (*P* = 0.551). There was one case of morbidity in a patient with a mRS of 4 in the SAC group and no mortality in either group.

## Discussion

While the indications for PED have been extended to include intracranial small and blister aneurysms in the anterior circulation (1, 12, 13), PED has also been applied in the treatment of posterior circulation aneurysms. However, the feasibility as well as the advantage of this treatment remain controversial, especially in that traditional endovascular treatment modalities have already demonstrated acceptable safety and efficacy profiles (14–16). Therefore, it is necessary to compare the treatment outcomes between PED and SAC for non-saccular, unruptured, intradural vertebral artery aneurysms of the posterior circulation.

The major concern with PED for posterior circulation aneurysms is its high overall morbidity and mortality. As reported in an IntrePED study by Kallmes et al. the rate of neurological morbidity and mortality was 16.4% for posterior circulation aneurysms, which was higher than the rate of neurological morbidity and mortality for anterior internal carotid artery aneurysms (95% CI, 6.5–10.3%; *P* = 0.01) (17). Lopes et al. further investigated this phenomenon in a subgroup analysis of the IntrePED study, with a median follow-up of 21.1 months; the authors found that the neurological morbidity and mortality rate was 13.2% in 91 patients with 95 aneurysms (18). Use of multiple PEDs or too many complex, large, or basilar aneurysms might contribute to the high overall morbidity and mortality.

A recently meta-analysis by Kiyofuji et al. also reported higher morbidity and mortality rates of 26 and 21%, respectively (19), but the authors found that the rate of favorable neurological outcomes in vertebral artery aneurysms was higher than that for aneurysms of the mid/holobasilar and vertebrobasilar junction, indicating that flow diverters could be safely used in the vertebral aneurysms. Some previous small studies have reported favorable outcomes with the use of PED to treat non-saccular, unruptured, intradural vertebral artery aneurysms (8–10). The authors report complete occlusion rates of 66.7–100%, without any morbidity and mortality, indicating that the PED might be safe and feasible for these aneurysms. However, the conclusions of these studies are not sufficiently validated, as they did not compare PED with traditional treatment, in particular the commonly used SAC, which has been deemed safe and effective for these aneurysms.

This is the first study to explore the feasibility of the PED for the treatment of non-saccular, unruptured, intradural vertebral artery aneurysms by comparing it with SAC. In the present study, we achieved good neurologic outcomes, though the SAC group was associated with a higher morbidity rate of 1.6% (*P* = 1). The peri-procedural complication rate of the PED group was 9.4%, which appeared to be higher than that for the SAC group, but this difference did not reach statistically significance (*P* = 0.397). All patients in the PED group were asymptomatic, with a mRS of 0–2 at discharge. The PED group had a higher technical event rate of 9.4% compared with the SAC group, but this difference was not statistically significant (*P* = 0.397). The procedure length was similar between the two groups (*P* = 0.386).

Late PAO is one of the most serious delayed complications associated with endovascular treatment devices. In the present study, the PED group showed a higher PAO rate of 6.7% than the SAC group, although this was not statistically significant (*P* = 0.625). Becske et al. reported 5 cases of PAO in their series studying PED (1). The authors concluded that noncompliance with antiplatelet medication and severe in-stent stenosis might lead to PAO and monitoring antiplatelet effectiveness might be useful to reduce the rate of this complication. Oishi et al. reported the long-term follow-up of a patient with PAO who was treated with PED in their institution (20). The authors found thrombus development at the non-covered part with endothelium due to discontinuation of antiplatelet therapy and incomplete occlusion of the aneurysm; this was previously described by Kadirvel et al. in an animal study of PED that investigated the cellular mechanisms of aneurysm occlusion (21). The authors concluded that antiplatelet therapy should not be stopped until complete occlusion of the aneurysm is detected, and they reported that multi-layer PED deployment might be a risk factor for PAO. In the present study, in-stent thrombosis associated with PAO developed in two cases in the PED group. This occurred due to in-stent stenosis (>50%) of the parent artery, which we summarized in our recently report (6). Performing balloon angioplasty or using stronger radial support stents with longer antiplatelet treatment and angiographic follow-up might be useful to reduce the rate of this complication.

Previous large studies reported a higher complete occlusion rate of aneurysms treated with SAC (range 59–79.8%) (14, 16). In the present study, we found a similar, favorable complete occlusion rate of 72.5% in the SAC group. However, we obtained a higher complete occlusion rate of 90% in the PED group. In addition, we found that aneurysms in the PED group required a shorter time to achieve this endpoint than aneurysms in the SAC group (*P* < 0.001). Multivariate analysis indicated that the aneurysms in the PED group were more prone to achieve this endpoint over time (*P* < 0.001). All the covered PICAs were patent in the PED group, which is similar to the outcomes reported in previous studies (9, 10, 22).

Smoking is considered to increase the risk of recanalization after endovascular treatment and is a risk factor for aneurysmal subarachnoid hemorrhage (23, 24). However, in a recently study by Adeeb et al., the authors found that patients with a smoking history had a higher rate of aneurysm occlusion, and they thought that smoking could promote intra-aneurysmal thrombosis (25). In a previous study by Rouchaud et al. the authors reported that smoking was not associated with the complete occlusion rate, major morbidity rate, and neurologic mortality rate of aneurysms (26). Similar to the findings of a long-term follow-up study on the impact of smoking by Brinjikji et al. the authors also found that smoking was not a risk factor for aneurysm recurrence and retreatment (27). In the present study, we found that smoking was a risk factor for aneurysm occlusion. This outcome may be attributed to the short follow-up interval and small sample size.

Taken together, similarly low peri-procedural and delayed complication rates and higher complete occlusion rate of the PED group compared with the SAC group suggest that the PED could be safe and effective for the treatment of non-saccular, unruptured, intradural vertebral artery aneurysms.

## Limitations

This is a retrospective study with a short follow-up interval and was performed at a single center. And the operations were mainly performed by three different interventionalists. Though all of them were experienced and skillful, there might be observational bias. We did not perform subgroup analysis in the SAC group to detect the differences in therapeutic efficiency among the three stent types (LVIS, Enterprise, and Neuroform). Because previous studies have demonstrated the safety and efficacy of these stents, in the present study, we aimed to compare these traditional stents with the PED. This study included two patients with bilateral vertebral aneurysms and four aneurysms treated with PED and additional coiling. Despite these limitations, this is the first study to explore the safety and efficiency of PED in non-saccular, untreated, intradural vertebral artery aneurysms by comparing well-matched PED and SAC groups.

## Conclusions

Both PED and SAC are safe and effective for the treatment of non-saccular, untreated, intradural vertebral artery aneurysms. The technical factors, procedural complications, angiographic results, and rate of favorable clinical outcomes were similar between the two therapeutic strategies. Aneurysms treated with PED were more prone to obliteration over time than were aneurysms treated with SAC. These results suggest that the PED is a safe and feasible strategy for the treatment of non-saccular, unruptured, intradural vertebral artery aneurysms. However, larger studies with long-term follow-up are needed to explore the optimal therapeutic strategy for these aneurysms.

## Ethics Statement

The study was approved by the Tiantan Hospital committee. All subjects gave written informed consent in accordance with the Declaration of Helsinki.

## Author Contributions

YpZ conception and design the article, and analysis and interpretation of data, and revise the article critically. FL acquisition of data, analysis and interpretation of data, and draft the article. YxZ acquisition of data, and give final approval of the version to be submitted. PY acquisition of data, and give final approval of the version to be submitted. SL give final approval of the version to be submitted. CM give final approval of the version to be submitted. CJ revise the article critically, and give final approval of the version to be submitted.

### Conflict of Interest Statement

The authors declare that the research was conducted in the absence of any commercial or financial relationships that could be construed as a potential conflict of interest.

## Abbreviations

PED: pipeline embolization device
SAC: stent-assisted coiling
HR: hazard ratio
CI: confidence interval
mRS: modified Rankin Scale
LVIS: Low-prole Visualized Intraluminal Support
PAO: Parent artery occlusion
PICA: posterior inferior cerebellar artery.
